# Adults social media addiction and phubbing latent profiles and authenticity as outcome

**DOI:** 10.1186/s40359-025-02858-y

**Published:** 2025-05-30

**Authors:** Ali Kirksekiz, Mehmet Yildiz, Mübin Kiyici, Mürüvvet Emrem, Sevgi Koroglu, Metin Yıldız

**Affiliations:** 1 Vocational School of Informatics Technologies, Sakarya University of Applied Sciences, Sakarya, Turkey; 2 Distance Education Research and Application Center, Sakarya University of Applied Sciences, Sakarya, Turkey; 3 Educational Sciences Instructional Technology Department, Sakarya University, Sakarya, Turkey; 4 Faculty of Health Sciences, Kocaeli University, Kocaeli, Turkey; 5 Faculty of Health Sciences, Sakarya University, Sakarya, Turkey

**Keywords:** Adults, Social media addiction, Authenticity, Phubbing, Latent profile analysis

## Abstract

This study was conducted to examine the effect of social media addiction and phubbing on authenticity in adults. This study based on person-centered approach and cross-sectional design, was conducted with 1046 individuals living in Turkiye between February and August 2024. R programming language 4.1.3, G*Power 3.1 and SPSS-22 program were used in the analysis of the study. With LPA it was found that there are 3 classes. It was found that individuals with high digital addiction had low levels of authenticity (*p* <.05). In our study, digital addiction was found to negatively affect the level of authenticity. It is recommended to examine the effect of digital addiction on autism and other psychological factors.

## Introduction

According to Statista 2025 approximately 67.9% of the global population, equating to 5.56 billion people, are internet users as of 2025 [1]. The rapid expansion of social networks, driven by the widespread use of smartphones and constant internet access, has made them an integral part of daily life for millions worldwide, increasing the risk of addictive behaviors [2]. Social media addiction is often defined by an uncontrollable urge that leads to excessive time consumption and inefficiency [3, 4].

The growing number of social media users is largely due to its various benefits, including providing social support, enabling easy access to information, and enhancing overall life satisfaction [4, 5]. However, despite these advantages, problematic social media use remains a global issue [6], contributing to psychological distress [7], excessive screen time [8], and a sedentary lifestyle [9] all of which have been linked to poorer mental health outcomes in certain populations [10]. This addiction negatively impacts social interactions, work performance, psychosocial well-being, and interpersonal relationships [3, 4]. Additionally, the motivations behind social media use play a crucial role in shaping users’ perceptions of authenticity.

Authenticity consists of two key components: authentic self-awareness and authentic self-expression [11]. he former refers to an individual’s ability to understand and explore their identity, while the latter—especially relevant in the context of social media—pertains to presenting oneself in a manner consistent with one’s self-perception [11]. Authenticity is considered essential for psychological well-being and has been linked to improved mental health outcomes [12]. Presenting oneself authentically on social media has been associated with positive psychological benefits, as it allows users greater control over self-expression [13]. Sharing spontaneous and informal aspects of daily life fosters better mental health [14], while authenticity in online interactions has been shown to predict greater life satisfaction, subjective well-being, positive emotions, and lower levels of depression [15–17].

Three primary factors contribute to social media addiction. The first is false self-presentation, where individuals alter their behavior or appearance to create a desired impression [18]. The second is FOMO (Fear of Missing Out), which refers to the anxiety of missing social interactions or opportunities to engage with peers [19]. The third factor is phubbing, a behavior where individuals prioritize their smartphones over in-person conversations, exhibiting antisocial tendencies [20].

Phubbing, a term derived from the words “phone” and “snubbing,” describes the habit of ignoring others in favor of using a smartphone during face-to-face interactions [21]. This behavior disrupts interpersonal communication, affecting relationships across various social groups and age demographics. Research indicates that phubbing contributes to psychological distress and weakens social connections [22]. Studies have also found that phubbing reduces conversational intimacy [21, 23], as individuals distracted by their devices struggle to engage in meaningful communication and behaviors that foster closeness [23]. Furthermore, its adverse effects extend beyond communication, impacting both the physical and psychological well-being of those who engage in or experience phubbing [21, 24].

Adolescents are increasingly incorporating social media into their daily lives while resisting parental restrictions. This life stage is characterized by emotional instability, increased risk-taking behaviors, and the onset of mental health disorders such as depression [10]. The aim of this study was to examine the implicit profiles of social media addiction and phubbing in adults and authenticity as a consequence.

### Hypotheses

#### H_0_

Social media addiction and phubbing are not effective on authenticity by latent profile analysis.

#### H_1_

Social media addiction and phubbing affect authenticity through latent profile analysis.

## Method

### Place and time of the research

This study based on person-centered approach and cross-sectional design, was conducted with 1046 individuals living in Turkiye between February and August 2024.

### Population and sample of the study

The population of the study consisted of individuals living in Turkey and aged 18 and over. The sample of the study was found to be 384 in the calculation made by the unknown sampling method. Following the study, a post hoc power analysis was conducted based on the results obtained from 1046 participants, revealing that the study’s power is 99% at a medium effect size and a 95% confidence level [25]. The STROBE guideline was used in the reporting of this research paper [26].

### Inclusion criteria

Individuals who met the criteria of the study, whose native language was Turkish, who were studying at the relevant university and who voluntarily agreed to participate in the study.

### Exclusion criteria

Those who left the questionnaire unfinished were not included in the study.

### Data collection

Participants were informed in the online form that the confidentiality of the data would be protected, that personal information would not be requested, that the study was voluntary and that they could terminate the study at any time. The individuals to be included in the study were recruited on the basis of their consent and voluntariness. Students who agreed to participate in the study were asked to fill in the data collection forms, which took approximately 15 min.

### Data collection tools

Data were collected using the ‘Introductory Information Form’, ‘Bergen Social Media Scale’, ‘General Phubbing Scale’, ‘Authenticity Scale’.

### Introductory information form

It consists of questions about the demographic characteristics of the individuals.

### Bergen social media addiction scale

Bergen Social Media Addiction Scale was developed by Andreassen et al. [27] Its adaptation in Turkish language was conducted by Demirci in 2019 [28]. This scale was used because it has been used in many countries around the world and has high reliability. The scale consists of a single dimension and six items. Each item in the scale meets six basic addiction criteria: mental occupation, mood change, tolerance, withdrawal, conflict and failed quit attempt. The scale is answered on a five-point Likert-type scale ranging from (1) very rarely to (5) quite often. The score that can be obtained from the scale varies between 6 and 30. There are no reverse items in the scale. An increase in the score on the scale indicates an increase in social media addiction. The internal consistency of the scale was found to be 0.83 [28]. In our study, Cronbach’s alpha value of the scale was found to be 0.87.

### General scale of phubbing (GSP)

This scale was first developed by Chotpitayasunondh and Douglas (2018a). The scale consists of 15 items and is measured on a seven-point Likert scale (1: Never, 7: Always). The scale consists of four subscales: Nomophobia (NP), Interpersonal Conflict (IC), Self-Isolation (SI) and Problem Acceptance (PA). The measure has a good α Internal Reliability (IR) of 0.85 to 0.92 and convergent validity [29]. Turkish adaptation was made by Ergün et al. [30]. Ergun et al. found the Cronbach’s alpha value of the scale to be 0.91 [30]. In our study, Cronbach’s alpha value of the scale was found to be 0.95.

### Authenticity scale

This scale was developed by Wood, Linley, Maltby, Baliousis, Joseph in 2008 to measure the authentic characteristics of individuals [31]. The scale consists of 3 dimensions: acceptance of external influence, self-alienation and authenticity of life. The reliability of the scale was found to be 0.94. It consists of 12 items in total, 4 items each as Self Alienation (2, 7, 10, 12), Acceptance of External Influences (3, 4, 5, 6) and Authentic Life (1, 8, 9, 11). The Likert-type of the scale is graded on a 7-point scale. A minimum of 12 and a maximum of 84 points are calculated from the scale. There are no reverse scored items in the scale. In our study, Cronbach’s alpha value of the scale was found to be 0.80.

### Data analyze

The analysis of the study data was performed by using SPSS 22.0, and G*Power 3.1 Statistical package software. Post hoc power analysis uses the information on effect size, sample size and significance level obtained after a study has been completed to calculate the statistical power of the test. This power is defined as 1 - β and indicates the probability that the study detected a true effect. Percentage, arithmetic mean, standard deviation, minimum and maximum values were calculated using SPSS 22.0. Necessary normality tests were performed in the process of analyzing the data and it was understood that the kurtosis and skewness of data between − 1.5 to + 1.5 [32]. P value of 0.05 was considered statistically significant. In the study, latent profile analysis was performed with R programming language 4.1.3. Latent Profile Analysis (LPA) is a statistical method used to identify different subgroups within a population. Latent profile analysis allows to uncover hidden underlying patterns based on observed data of individuals. This method reveals subgroups by analyzing the patterns of individuals’ responses to various observable variables [33]. This method is particularly useful for understanding a heterogeneous community. By examining various characteristics, attitudes, behaviors or responses of individuals to certain variables, LPA aims to determine which subgroup these individuals belong to within a larger community. This analysis treats each individual independently and classifies them into groups based on common characteristics. For example, it can be used to identify subgroups in a group of students based on their learning styles, motivation levels or academic achievement. Thus, the differences between individuals and the underlying reasons for these differences can be better understood, rather than the overall characteristics of the community. LPA can be applied in many fields such as health, education and psychology and provides important information for developing more targeted strategies in decision-making processes. It is a powerful method to explore the more complex structure of a community based on individual differences [34]. Rather than treating the population as a single entity, LPA identifies subgroups or profiles that bring together similar individuals by examining differences in the behavior, attitudes or characteristics of individuals. The main goal of LPA is to uncover hidden structures that exist within a larger population by finding similarities and differences between individuals’ responses or patterns of behavior. By identifying which group individuals belong to, this method makes it easier to understand each group’s unique needs, strengths and areas for improvemen [34–37]. Through their hidden profiles, researchers can develop a deeper understanding of complex phenomena such as motivation, social media use, emotional labor strategies and online gaming addiction [33, 35, 38]. Mclust library [39], was used for LPA analysis and Tidylpa library [40] was used to access fit indices during the reporting phase. In model selection, statistical fit criteria such as AIC (Akaike Information Criterion) and BIC (Bayesian Information Criterion) guide the determination of the best number of profiles. These criteria balance the fit and complexity of the model, helping to select models that explain better with fewer parameters.

## Results

The first step of LPA analysis is to decide on the model.

At this stage, BIC values were obtained by iterating each model and each class for 4 models and 3 classes. Akogul & Erisoglu (2017) stated that other fit indices can also be considered in the evaluation step of the models [41]. However, Tein et al. (2013) stated in their review that BIC is the most widely used evaluation criterion in the literature [42]. BIC values are shown in Table 1.

**Fig. 1:**
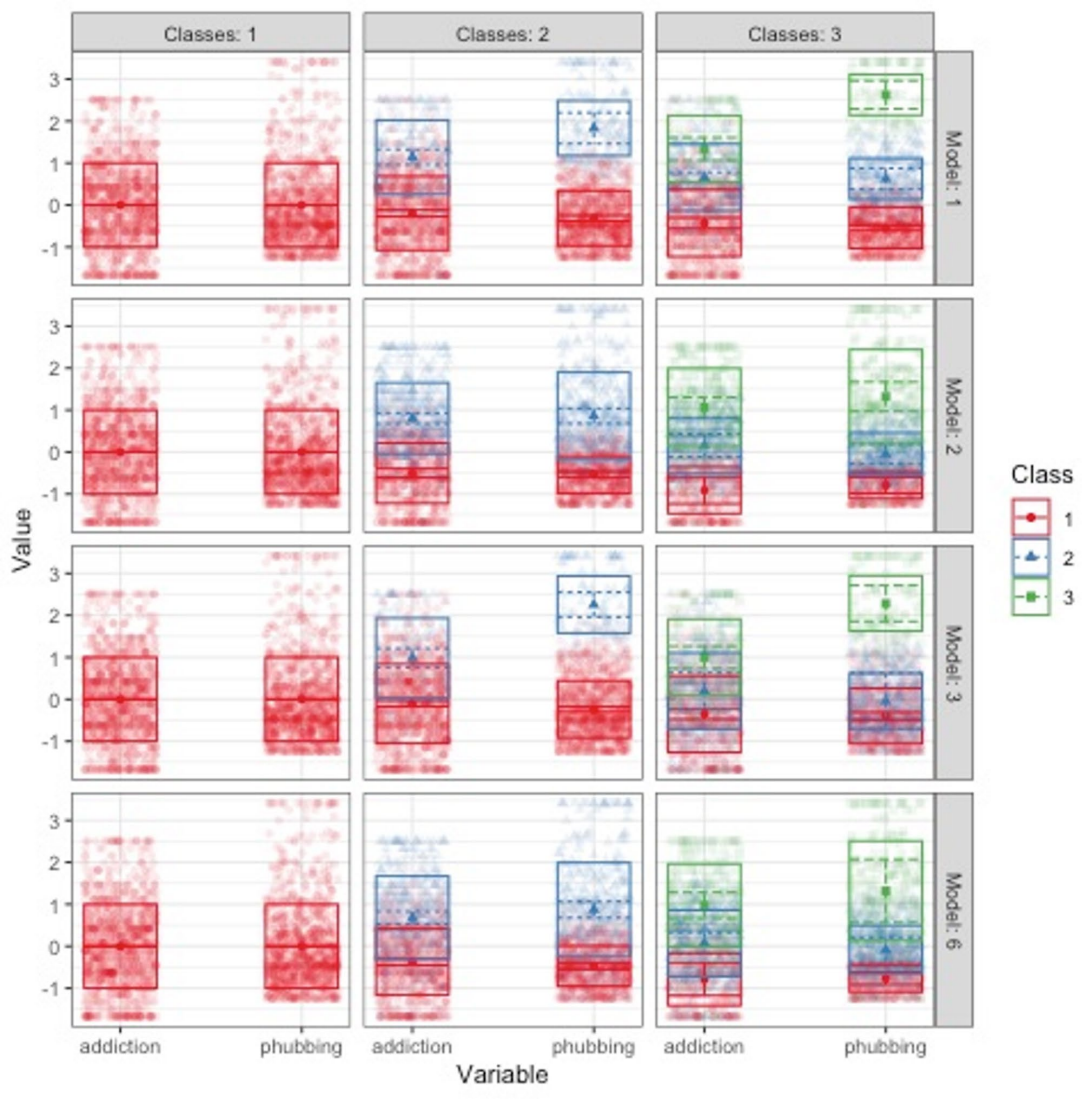
BIC values

The representation of each class up to 4 classes for EEI, VVI, EEE, VVV models is shown in Fig. 1.

**Table 1:** BIC values of all models

Class	EEI	VVI	EEE	VVV
1	-15818,00084	-15818,00084	-15386,88904	-15386,88904
2	-15368,11296	-15192,14551	-15105,91576	-15004,80653
3	-15161,21777	-15029,15014	-15128,92359	-14967,83397

**Fig. 2:**
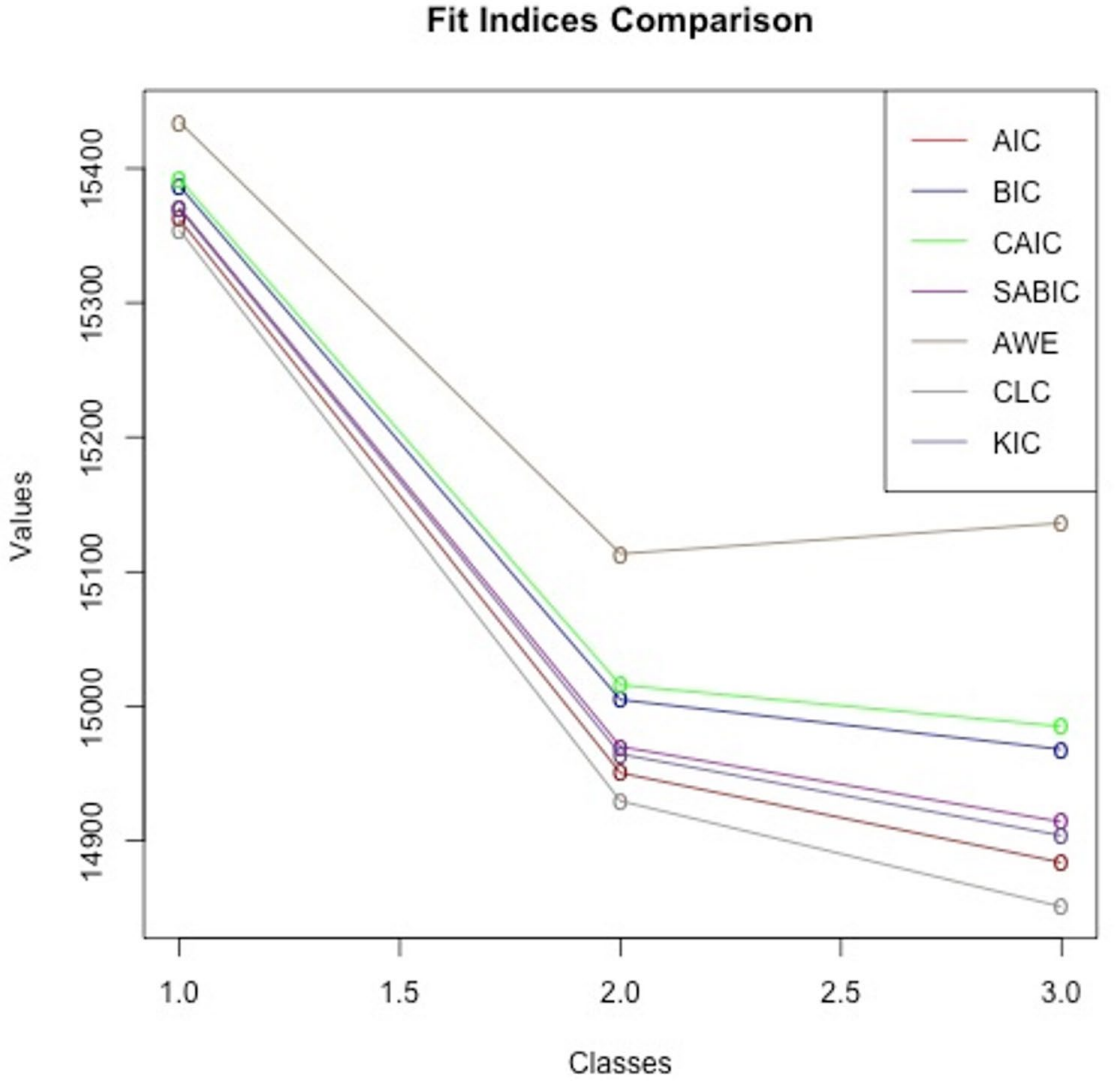
Elbow plots of AIC, BIC, CAIC, SABIC, AWE, CLC and KIC values

Elbow plots of AIC, BIC, CAIC, SABIC, AWE, CLC and KIC values for deciding the number of classes after determining the VVV model as the most appropriate are shown in Fig. 2 shows.

**Fig. 3:**
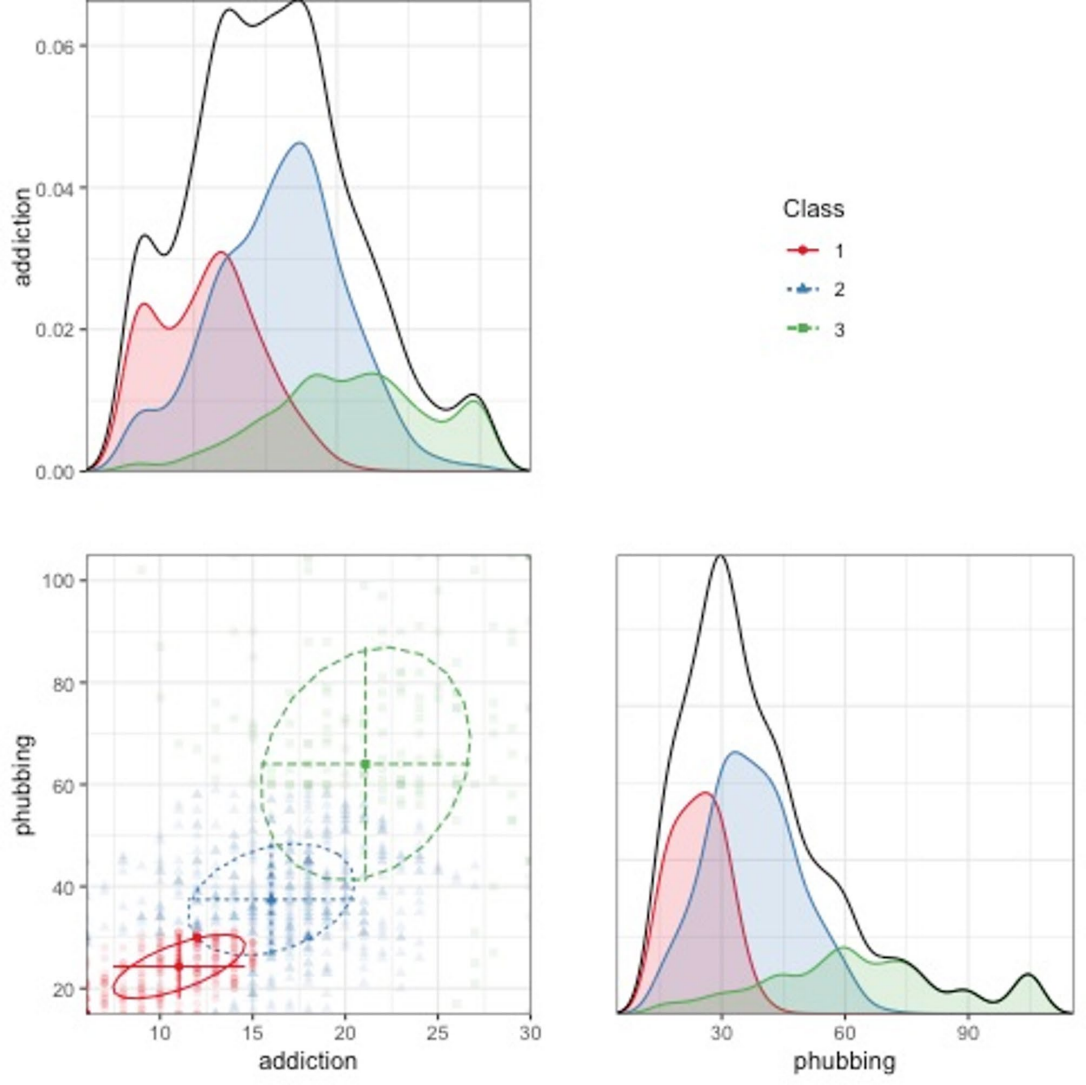
Density and uncertainty distributions of classes

Figure 2 When the elbow plots are examined, the lowest value according to the fit indices or the decrease in the slope of the decrease after a certain slope decrease is one of the criteria used to determine the number of classes. Low values of BIC and AIC indicate that the best model fit is realized [43]. Table 2 shows the extra indices and values used in the model evaluation. Figure 3; Tables 2 and 3, it is concluded that the best fitting class is the 3-class solution.

**Table 2:** Extra indices and values used in model evaluation

Model	Classes	LogLik	AIC	AWE	BIC	CAIC	CLC	KIC	SABIC	ICL	Entropy	LMR_val	LMR_p	LMR_mean
6	1	-7676,062	15362,125	15434,652	15386,889	15391,889	15354,125	15370,125	15371,008	-15386,889	-	-	-	-
6	2	-7464,1632	14950,326	15113,168	15004,806	15015,806	14929,444	14964,326	14969,868	-15315,968	423.798	0.001	1 < 2	423.798
6	3	-7424,818	14883,637	15135,984	14967,833	14984,833	14850,683	14903,637	14913,839	-15544,927	78.688	0.001	2 < 3	78.688

**Table 3:** Class classification

Classes	1	2	3	Total
1	1046 (1)			1046
2	739 (0.71)	307 (0.29)		1046
3	324 (0.31)	551(0.53)	171(0.16)	1046

**Fig. 4:**
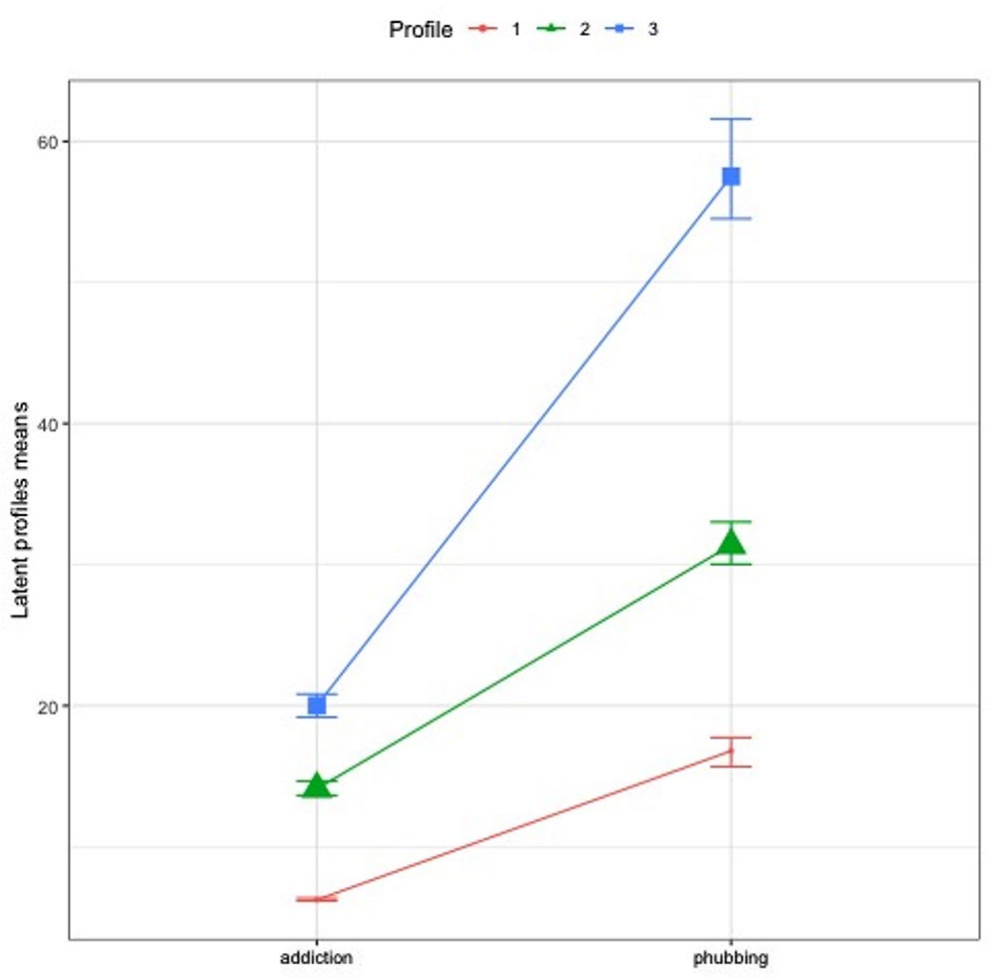
Line graph of indicators’ means by classes

**Fig. 5:**
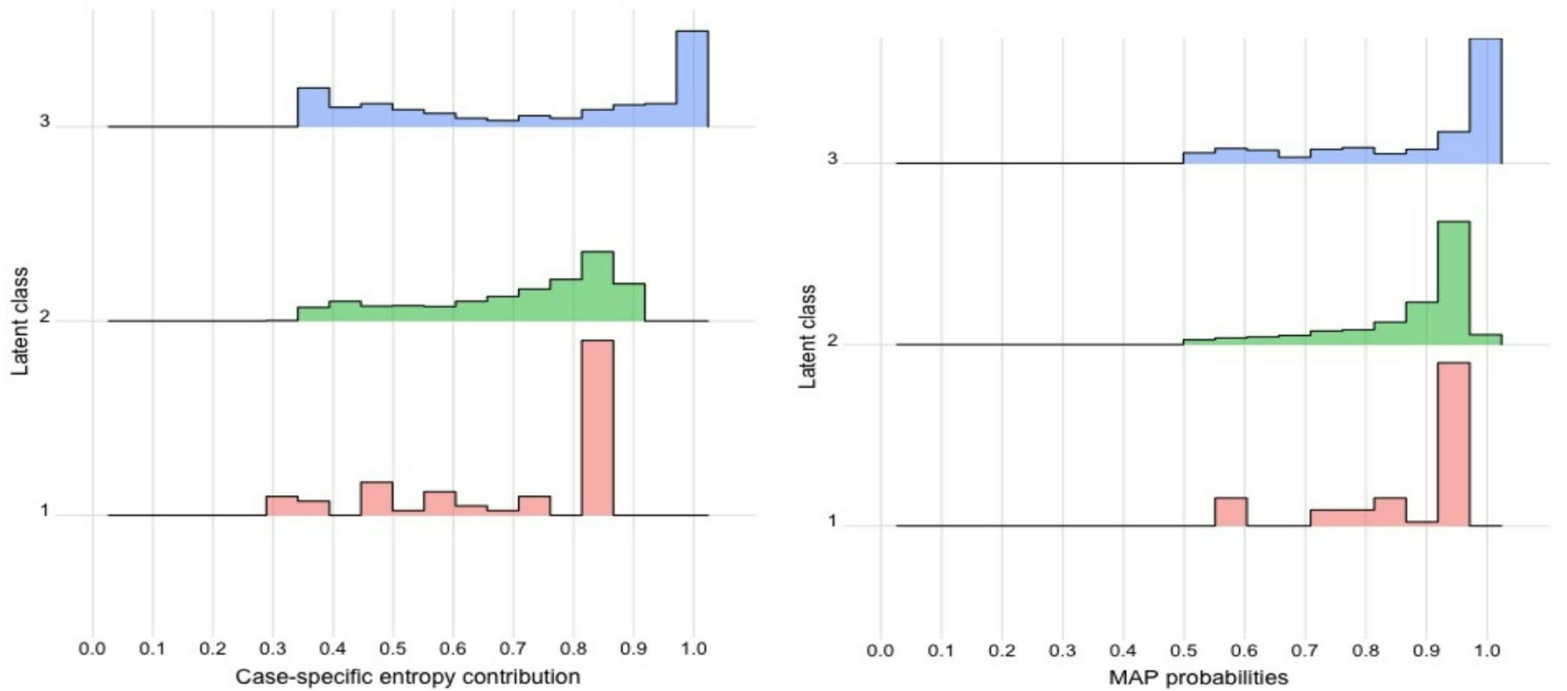
Entropy and MAP values

Tables 2 and 3 show the class sizes and class averages. Figure 4 shows the line graphs of indicators’ means by classes. As a result of LPA, class 3 has the highest arithmetic mean for all indicators. Case-specific entropy contribution and (Maximum A Posteriori) MAP values are given in Fig. 5.

In the post hoc analysis (Games Howell) conducted according to the latent classes of individuals in our study, it was found that the authenticity level of individuals with high digital addiction was significantly lower than the individuals in the other group (*p* <.05) (Tables 3 & 4).

**Table 4:** Comparison of individuals’ latent classes and authenticity scale mean scores (*N* = 1046)

			Authenticity scale	
		*n*	$\overline{X}$±SD	Test and significance
Latent Classes	Class 1	64	63.39 ± 13.81	F = 49.080 *p* =.001
	Class 2	691	61.94 ± 12.01	
	Class 3 (High digital addiction)	291	53.87 ± 11.65	
η² ≈ 0.086				

* One-Way ANOVA test

## Discussion

The aim of this study was to examine the implicit profiles of social media addiction and phubbing in adults and authenticity as a consequence. In this part of the study, the findings are discussed in the light of the literature.

In our study, in the post hoc analysis (Games Howell) conducted according to the latent classes of individuals, it was found that the authenticity level of individuals with high digital addiction was significantly lower than the individuals in the other group (*p* <.05). Social media addiction is increasingly attracting attention as a phenomenon that negatively affects individuals’ real-life social interactions and authenticity levels. Studies show that social media addiction negatively affects individuals’ social skills, emotional intelligence levels and overall life satisfaction. Especially among young people, social media addiction leads to weakened interpersonal relationships and lower levels of empathy [44–46].

Although social media addiction increases individuals’ search for social support, it has been revealed that this search causes weakening in family and friend relationships. In Yıldız and Koçak’s study, it was stated that social media addiction increases with the increase in the duration of social media use and this increases the need for emotional support [45]. This situation causes individuals to move away from real social relationships and thus decreases their level of authenticity [44, 45].

The relationship between social media addiction and authenticity is a complex interplay that has garnered significant attention in recent research. Social media addiction, characterized by excessive engagement with social media platforms, has been shown to negatively impact individuals’ mental health and self-esteem, which in turn affects their perceived authenticity in online interactions. For instance, Hou et al. (2019) found that social media addiction is associated with reduced mental health, primarily through its detrimental effect on self-esteem [47].

This decline in self-esteem can lead individuals to present curated versions of themselves online, further complicating their authenticity. The motivations behind social media use can influence the degree of authenticity perceived by users. Reinecke and Trepte (2014) reported that individuals who presented their true selves on Facebook experienced higher levels of positive affect and lower levels of negative affect. Similarly, Grieve and Watkinson (2016) demonstrated that individuals who were more authentic on social networking sites (e.g., Facebook) experienced greater social connectedness and reduced stress [15, 17].

Altayef & Karacı (2019) highlighted that students using social media platforms for social interaction often experience higher levels of addiction, which correlates with attention deficits and lower academic performance [48]. This suggests that the compulsive nature of social media use may lead individuals to prioritize engagement over genuine self-presentation, thereby undermining their authenticity.

The concept of authenticity itself is multifaceted, encompassing both existential and object-based dimensions. Zhu et al. (2023) discussed how social media can enhance perceptions of authenticity through rich experiential features, which can influence users’ psychological mechanisms [49]. However, platforms like BeReal, which claim to promote authenticity, have sparked debates about the extent to which social media can genuinely facilitate authentic interactions. Reddy (2023) noted that while users may perceive certain platforms as authentic, there remains skepticism about the overall authenticity of social media as a medium [50].

The constant need for self-expression and approval of details on digital platforms diminishes individuals’ capacity to explore the deeper layers of their subjective self [51]. The digital space tends to prioritize instantaneous and data-driven communication over meaningful and enduring relationships developed over time [52]. This undermines efforts to build a resilient self on solid foundations and instead fosters a self-presentation shaped by social validation and popular culture dynamics [51]. Additionally, digital relationships draw individuals away from real-life experiences and face-to-face interactions, which compromises self-coherence and the expression of authenticity, while strengthening performative self-presentations [52, 53].

Another key dimension is the role of social media influencers, who often curate seemingly authentic personas. However, this performance can result in a paradox wherein authenticity is carefully constructed. Balaban and Szambolics (2022) argue that authenticity on social media is closely tied to perceived trust and originality, yet the widespread use of filters and digital editing undermines this perception [54]. This tension reveals the inherent contradiction between the ideal of authenticity and the reality of curated online identities.

In conclusion, the relationship between social media addiction and authenticity is characterized by a cycle where addiction undermines self-esteem and genuine self-presentation, while the constructed nature of online identities complicates perceptions of authenticity. As social media continues to evolve, understanding this dynamic will be essential for both users and researchers alike.

Phubbing, a term derived from the words “phone” and “sneer”, refers to the act of ignoring someone in favor of using a mobile device. This behavior has become prominent with the rise of smartphones and has significant implications for social interactions and relationships. The relationship between increased phubbing and decreased authenticity in interpersonal interactions is multifaceted, involving psychological, social and emotional dimensions. Research shows that phubbing can lead to feelings of discomfort and neglect among those being phubbed, which can threaten the quality of social interactions and relationships [55]. This phenomenon is particularly evident in romantic relationships, where partner phubbing can evoke feelings of jealousy and insecurity and ultimately reduce relationship satisfaction [56, 57]. The emotional disconnect caused by phubbing can erode the authenticity of interactions as individuals may feel less valued and more isolated in their relationships [58]. Moreover, the psychological underpinnings of phubbing reveal a correlation with anxiety and social discomfort. Individuals with higher levels of personality anxiety are potentially more prone to engage in phubbing behaviors as a maladaptive coping mechanism to alleviate their discomfort in social situations [59, 60]. This suggests that the act of phubbing may reflect not only a lack of attention to the current social context, but also a deeper struggle with personal emotional regulation, further distancing from genuine interaction with others [61]. The impact of phubbing extends beyond individual relationships; it can also disrupt family ties and parent-child interactions. Studies have shown that parental phubbing can lead to social disconnection and negatively affect children’s emotional well-being, reinforcing the idea that phubbing contributes to a broader social malaise characterized by reduced authenticity in familial relationships [62, 63]. This disconnect may be due to a perception of being devalued or ignored, which can hinder the development of secure attachments and authentic communication within families [64]. In addition to emotional and relational consequences, increased phubbing is also linked to addictive behaviors associated with social media and mobile device use. Fear of missing out (FOMO) and problematic social media use have been identified as significant predictors of phubbing behavior, suggesting that individuals may prioritize online interactions over face-to-face connections [65, 66]. This prioritization can lead to a cycle where the authenticity of social interactions is further compromised as individuals become immersed in their devices rather than the meaningful exchanges taking place around them [67].

## Conclusion

In our study, according to the latent classes of individuals, it was found that the authenticity level of individuals with high digital addiction was significantly lower than the individuals in the other group. Longitudinal studies on the effects of addiction are recommended.

### Study limitations

The limitation of the cross-sectional study is that it reflects the results of the period in which it was conducted since it was conducted in a certain period of time.

## Data Availability

The data is available upon request from the corresponding author.

## References

[1] Petrosyan A. Number of internet and social media users worldwide as of February 2025. Retrieved from Statista database: https://www.statista.com/statistics/617136/digital-population-worldwide/

[2] Huang P-C, Latner JD, O’Brien KS, Chang Y-L, Hung C-H, Chen J-S, Lee K-H, Lin C-Y. Associations between social media addiction, psychological distress, and food addiction among Taiwanese university students. J Eat Disorders. 2023;11(1):43. 10.1186/s40337-023-00769-0 PMC10031987 36945011

[3] Cheng C, Lau Y-c, Chan L, Luk JW. Prevalence of social media addiction across 32 nations: Meta-analysis with subgroup analysis of classification schemes and cultural values. Addict Behav. 2021;117:106845. 33550200 10.1016/j.addbeh.2021.106845

[4] Mitropoulou EM, Karagianni M, Thomadakis C. Social media addiction, Self-Compassion, and psychological Well-Being: A structural equation model. Alpha Psychiatry. 2022;23(6):298. 36628380 10.5152/alphapsychiatry.2022.22957 PMC9797840

[5] Sheldon P, Antony MG, Sykes B. Predictors of problematic social media use: personality and life-position indicators. Psychol Rep. 2021;124(3):1110–33. 32580682 10.1177/0033294120934706

[6] Stănculescu E, Griffiths MD. The association between problematic internet use and hedonic and Eudaimonic well-being: A latent profile analysis. Technol Soc. 2024;78:102588.

[7] Geirdal AØ, Ruffolo M, Leung J, Thygesen H, Price D, Bonsaksen T, Schoultz M. Mental health, quality of life, wellbeing, loneliness and use of social media in a time of social distancing during the COVID-19 outbreak. A cross-country comparative study. J Mental Health. 2021;30(2):148–55. 10.1080/09638237.2021.1875413 33689546

[8] Sümen A, Evgin D. Social media addiction in high school students: a cross-sectional study examining its relationship with sleep quality and psychological problems. Child Indic Res. 2021;14(6):2265–83. 34367373 10.1007/s12187-021-09838-9 PMC8329411

[9] Sofiany IR, Setyawati MI. Portrait of the sedentary lifestyle among students from public health school. Muhammadiyah J Epidemiol. 2021;1(1):65–72.

[10] Valkenburg PM, Meier A, Beyens I. Social media use and its impact on adolescent mental health: an umbrella review of the evidence. Curr Opin Psychol. 2022;44:58–68. 34563980 10.1016/j.copsyc.2021.08.017

[11] Knoll M, Meyer B, Kroemer NB, Schröder-Abé M. It takes two to be yourself. J Individual Differences 2015.

[12] Smallenbroek O, Zelenski JM, Whelan DC. Authenticity as a Eudaimonic construct: the relationships among authenticity, values, and Valence. J Posit Psychol. 2017;12(2):197–209.

[13] Meier A, Reinecke L. Social media and mental health: Reviewing effects on eudaimonic well-being. 2021.

[14] Vromen A, Loader B, Manning N, Penfold-Mounce R, Xenos M. Politicians, celebrities and social media: a case of informalisation? 2017.

[15] Grieve R, Watkinson J. The psychological benefits of being authentic on Facebook. Cyberpsychology Behav Social Netw. 2016;19(7):420–5. 10.1089/cyber.2016.0010 27428029

[16] Mun IB, Kim H. Influence of false self-presentation on mental health and deleting behavior on Instagram: the mediating role of perceived popularity. Front Psychol. 2021;12:660484. 33912119 10.3389/fpsyg.2021.660484 PMC8071929

[17] Reinecke L, Trepte S. Authenticity and well-being on social network sites: A two-wave longitudinal study on the effects of online authenticity and the positivity bias in SNS communication. Comput Hum Behav. 2014;30:95–102.

[18] Michikyan M, Dennis J, Subrahmanyam K. Can you guess who I am? Real, ideal, and false self-presentation on Facebook among emerging adults. Emerg Adulthood. 2015;3(1):55–64.

[19] Roberts JA, David ME. Boss phubbing, trust, job satisfaction and employee performance. Pers Indiv Differ. 2020;155:109702.

[20] Karadağ E, Tosuntaş ŞB, Erzen E, Duru P, Bostan N, Şahin BM, Çulha İ, Babadağ B. Determinants of phubbing, which is the sum of many virtual addictions: A structural equation model. J Behav Addictions. 2015;4(2):60–74. 10.1556/2006.4.2015.005 PMC4500886 26014669

[21] Roberts JA, David ME. My life has become a major distraction from my cell phone: partner phubbing and relationship satisfaction among romantic partners. Comput Hum Behav. 2016;54:134–41.

[22] Karadağ E, Tosuntaş ŞB, Erzen E, Duru P, Bostan N, Şahin BM, Çulha İ, Babadağ B. The Virtual World’s Current Addiction: Phubbing. Addicta: Turkish J Addictions 2016, 3(2).

[23] Abeele MMV, Hendrickson AT, Pollmann MM, Ling R. Phubbing behavior in conversations and its relation to perceived conversation intimacy and distraction: an exploratory observation study. Comput Hum Behav. 2019;100:35–47.

[24] Davey S, Davey A, Raghav SK, Singh JV, Singh N, Blachnio A, Przepiórkaa A. Predictors and consequences of phubbing among adolescents and youth in India: an impact evaluation study. J Family Community Med. 2018;25(1):35–42. 29386960 10.4103/jfcm.JFCM_71_17 PMC5774041

[25] Cohen J. The concepts of power analysis. Stat Power Anal Behav Sci. 1988;2:1–17.

[26] Vandenbrouckel JP, von Elm E, Altman DG, Gotzsche PC, Mulrow CD, Pocock SJ, Poole C, Schlesselman JJ, Egger M. Strengthening the Reporting of Observational Studies in Epidemiology (STROBE): explanation and elaboration, PLoS Medicine, vol. 4, no. 10, Oct. 2007, p. 1628+. Gale OneFile: Health and Medicine. *PLoS Medicine* 2007, 4(10):1628–1655. http://link.gale.com/apps/doc/A171772985/HRCA?u=anon~7ce6cd36%26sid=googleScholar%26xid=cbcd6034. Accessed 2 Sept. 2024. 10.1371/journal.pmed.0040297 PMC2020496 17941715

[27] Andreassen CS, Pallesen S, Griffiths MD. The relationship between addictive use of social media, narcissism, and self-esteem: findings from a large National survey. Addict Behav. 2017;64:287–93. 27072491 10.1016/j.addbeh.2016.03.006

[28] Demirci İ. Bergen Sosyal Medya Bağımlılığı Ölçeğinin Türkçeye Uyarlanması, depresyon ve Anksiyete belirtileriyle Ilişkisinin değerlendirilmesi. Anadolu Psikiyatri Dergisi. 2019;20:15–22.

[29] Chotpitayasunondh V, Douglas KM. Measuring phone snubbing behavior: Development and validation of the Generic Scale of Phubbing (GSP) and the Generic Scale of Being Phubbed (GSBP). *Computers in human behavior* 2018, 88:5–17.

[30] Ergün N, Göksu İ, Sakız H. Effects of phubbing: relationships with psychodemographic variables. Psychol Rep. 2020;123(5):1578–613. 31752605 10.1177/0033294119889581

[31] Wood AM, Linley PA, Maltby J, Baliousis M, Joseph S. The authentic personality: A theoretical and empirical conceptualization and the development of the authenticity scale. J Couns Psychol. 2008;55(3):385.

[32] Tabachnick BG, Fidell LS, Ullman JB. Using multivariate statistics. Volume 6. pearson Boston, MA; 2013.

[33] Gabriel AS, Daniels MA, Diefendorff JM, Greguras GJ. Emotional labor actors: a latent profile analysis of emotional labor strategies. J Appl Psychol. 2015;100(3):863. 25068812 10.1037/a0037408

[34] Spurk D, Hirschi A, Wang M, Valero D, Kauffeld S. Latent profile analysis: A review and how to guide of its application within vocational behavior research. J Vocat Behav. 2020;120:103445.

[35] Lindwall M, Ivarsson A, Weman-Josefsson K, Jonsson L, Ntoumanis N, Patrick H, Thøgersen-Ntoumani C, Markland D, Teixeira P. Stirring the motivational soup: within-person latent profiles of motivation in exercise. Int J Behav Nutr Phys Activity. 2017;14(1):1–12. 10.1186/s12966-017-0464-4 PMC5237570 28088208

[36] Notelaers G, Einarsen S, De Witte H, Vermunt JK. Measuring exposure to bullying at work: the validity and advantages of the latent class cluster approach. Work Stress. 2006;20(4):289–302.

[37] Nguyen TTP, Do HN, Vu TBT, Vu KL, Nguyen HD, Nguyen DT, Do HM, Nguyen NTT, La LTB, Doan LP. Association of individual and neighborhood characteristics to problematic internet use among youths and adolescents: evidence from Vietnam. Int J Environ Res Public Health. 2023;20(3):2090. 36767455 10.3390/ijerph20032090 PMC9915430

[38] Demetrovics Z, Urbán R, Nagygyörgy K, Farkas J, Griffiths MD, Pápay O, Kökönyei G, Felvinczi K, Oláh A. The development of the problematic online gaming questionnaire (POGQ). PLoS ONE. 2012;7(5):e36417. 22590541 10.1371/journal.pone.0036417 PMC3349662

[39] Scrucca L, Fraley C, Murphy TB, Raftery AE. Model-based clustering, classification, and density Estimation using Mclust in R. Chapman and Hall/CRC; 2023.

[40] Rosenberg JM, Beymer PN, Anderson DJ, Van Lissa C, Schmidt JA. TidyLPA: an R package to easily carry out latent profile analysis (LPA) using open-source or commercial software. J Open Source Softw. 2019;3(30):978.

[41] Akogul S, Erisoglu M. An approach for determining the number of clusters in a model-based cluster analysis. Entropy. 2017;19(9):452.

[42] Tein J-Y, Coxe S, Cham H. Statistical power to detect the correct number of classes in latent profile analysis. Struct Equation Modeling: Multidisciplinary J. 2013;20(4):640–57. 10.1080/10705511.2013.824781 PMC3904803 24489457

[43] Ferguson SL, Moore G, Hull EW. Finding latent groups in observed data: A primer on latent profile analysis in Mplus for applied researchers. Int J Behav Dev. 2020;44(5):458–68.

[44] Akçay Bekiroğlu H, Şahin E. Kişisel beceriler, Kişilerarasi beceriler ve Genel Ruh Durumu Ile Sosyal Medya bağimliliği Ilişkisinin Üniversite öğrencileri örnekleminde analizi. J Marmara Univ Social Sci Institute/Öneri 2022, 17(58).

[45] Yıldız E, Koçak O. Üniversite öğrencilerinde Sosyal Medya bağimliliği ve Algilanan Sosyal Destek Arasindaki Ilişkinin değerlendirilmesi. Toplum Ve Sosyal Hizmet. 2020;31(3):1102–26.

[46] Çat AK, Koçak MC, Toprak Ö. Sosyal Medya Bağımlılık Düzeyinin Yaşam memnuniyetine Etkisi Üzerine Saha Araştırması. İletişim Kuram Ve Araştırma Dergisi. 2021;2021(56):185–202.

[47] Hou Y, Xiong D, Jiang T, Song L, Wang Q. Social media addiction: its impact, mediation, and intervention. Cyberpsychology: J Psychosocial Res Cyberspace 2019, 13(1).

[48] Altayef HAA, Karacı A. Analysis of social media addiction and usage purposes among secondary-school students in Turkey. AJIT-e: Acad J Inform Technol. 2019;10(38):7–34.

[49] Zhu C, Fong LHN, Liu CYN, Song H. When social media Meets destination marketing: the mediating role of attachment to social media influencer. J Hospitality Tourism Technol. 2023;14(4):643–57.

[50] Reddy A, Kumar P. Exploring authencity on the social media app bereal. AoIR Sel Papers Internet Res 2023.

[51] Mulyani M, Suwar A, Athal T. Challenges and strategies for maintaining Self-Authenticity in the digital age:: A philosophical and social analysis. Jurnal Ilmiah Teunuleh. 2024;5(3):111–26.

[52] Davis JL. Authenticity, digital media, and person identity verification. In: *Identities in everyday life.* edn.; 2019: 93–111.

[53] Nguyen L, Barbour K. Selfies as expressively authentic identity performance. First Monday 2017.

[54] Balaban DC, Szambolics J. A proposed model of self-perceived authenticity of social media influencers. Media Communication. 2022;10(1):235–46.

[55] Büttner CM, Gloster AT, Greifeneder R. Your phone ruins our lunch: attitudes, norms, and valuing the interaction predict phone use and phubbing in dyadic social interactions. Mob Media Communication. 2022;10(3):387–405.

[56] David ME, Roberts JA. Investigating the impact of partner phubbing on romantic jealousy and relationship satisfaction: the moderating role of attachment anxiety. J Social Personal Relationships. 2021;38(12):3590–609.

[57] Çizmeci E. Disconnected, though satisfied: phubbing behavior and relationship satisfaction. Turkish Online J Des Art Communication. 2017;7(2):364–75.

[58] Solecki S. The phubbing phenomenon: the impact on parent-child relationships. J Pediatr Nurs. 2022;62:211–4. 34629227 10.1016/j.pedn.2021.09.027

[59] Guazzini A, Duradoni M, Capelli A, Meringolo P. An explorative model to assess individuals’ phubbing risk. Future Internet. 2019;11(1):21.

[60] Parmaksız İ. Sosyotelizm (Phubbing) Ile Bilinçli Farkındalık Arasındaki Ilişkide Sosyal Kaygının Aracı Rolü. İnönü Üniversitesi Eğitim Fakültesi Dergisi. 2021;22(2):1387–420.

[61] Han JH, Park S-J, Kim Y. Phubbing as a millennials’ new addiction and relating factors among nursing students. Psychiatry Invest. 2022;19(2):135. 10.30773/pi.2021.0163 PMC8898607 35164436

[62] Pancani L, Gerosa T, Gui M, Riva P. Mom, dad, look at me: the development of the parental phubbing scale. J Social Personal Relationships. 2021;38(2):435–58.

[63] Liu K, Chen W, Lei L. Linking parental phubbing to adolescent self-depreciation: the roles of internal attribution and relationship satisfaction. J Early Adolescence. 2021;41(8):1269–83.

[64] Zhang J, Dong C, Jiang Y, Zhang Q, Li H, Li Y. Parental phubbing and child social-emotional adjustment: A meta-analysis of studies conducted in China. Psychol Res Behav Manage. 2023;4267:4285. 10.2147/PRBM.S417718 PMC10591670 37877136

[65] Franchina V, Vanden Abeele M, Van Rooij AJ, Lo Coco G, De Marez L. Fear of missing out as a predictor of problematic social media use and phubbing behavior among Flemish adolescents. Int J Environ Res Public Health. 2018;15(10):2319. 30360407 10.3390/ijerph15102319 PMC6211134

[66] Lv S, Wang H. Cross-lagged analysis of problematic social media use and phubbing among college students. BMC Psychol. 2023;11(1):39. 36765384 10.1186/s40359-023-01062-0 PMC9912680

[67] Karaman HB, Arslan C. The mediating role of social media addiction and phubbing in basic psychological needs in relationships and relationship satisfaction. Front Psychol. 2024;15:1291638. 38586290 10.3389/fpsyg.2024.1291638 PMC10995373

